# Use of timelapse photography to determine flower opening time and pattern in banana (*Musa* spp.) for efficient hand pollination

**DOI:** 10.1038/s41598-021-98500-z

**Published:** 2021-09-30

**Authors:** Allan Waniale, Rony Swennen, Settumba B. Mukasa, Arthur K. Tugume, Jerome Kubiriba, Wilberforce K. Tushemereirwe, Brigitte Uwimana, Gil Gram, Delphine Amah, Robooni Tumuhimbise

**Affiliations:** 1 Department of Agricultural Production, College of Agricultural and Environmental Sciences, Makerere University, P.O. Box 7062, Kampala, Uganda; 2 National Agricultural Research Laboratories, Kawanda, P.O. Box 7065, Kampala, Uganda; 3 International Institute of Tropical Agriculture, P.O. Box 7878, Kampala, Uganda; 4 Department of Biosystems, KU Leuven, W. De Croylaan 42, 3001 Heverlee, Belgium; 5 Department of Plant Sciences, Microbiology and Biotechnology, College of Natural Sciences, Makerere University, P.O. Box 7062, Kampala, Uganda; 6 International Institute of Tropical Agriculture, PMB 5320, Ibadan, 200001 Nigeria; 7 Rwebitaba Zonal Agricultural Research and Development Institute, P.O. Box 96, Fort Portal, Uganda

**Keywords:** Ecology, Plant sciences

## Abstract

Sterility and low seed set in bananas is the main challenge to their conventional genetic improvement. The first step to seed set in a banana breeding program depends on pollination at the right time to ensure effective fertilization. This study aimed at determining bract opening time (BOT) to enhance efficient pollination and seed set in bananas. A Nikon D810 digital camera was set-up to take pictures of growing banana inflorescences at five-minute intervals and time-lapse movies were developed at a speed of 30 frames per second to allow real-time monitoring of BOT. Genotypes studied included wild banana (1), Mchare (2), Matooke (4), Matooke hybrid (1), and plantain (1). Events of bract opening initiated by bract lift for female flowers (*P* < 0.01) started at 16:32 h and at 18:54 h for male flowers. Start of bract rolling was at 18:51 h among female flowers (*P* < 0.001) and 20:48 h for male flowers. Bracts ended rolling at 02:33 h and 01:16 h for female and flowers respectively (*P* < 0.05). Total time of bract opening (from lift to end of rolling) for female flowers was significantly longer than that of male flowers (*P* < 0.001). On average, the number of bracts subtending female flowers opening increased from one on the first day, to between one and four on the fourth day. The number regressed to one bract on day eight before start of opening of bracts subtending male flowers. There was a longer opening interval between bracts subtending female and male flowers constituting spatial and temporal separation. Bract rolling increased from partial to complete rolling from proximal to the distal end of the inflorescence among female flower. On the other hand, bracts subtending male flowers completely rolled. Differences in BOT of genotypes with the same reference time of assessment may be partly responsible for variable fertility. Hand pollination time between 07:00 and 10:00 h is slightly late thus an early feasible time should be tried.

## Introduction

Banana (*Musa* spp.) plants are monoecious where male and female flowers are primarily unisexual and separated on the same inflorescence^1^. They have a year-round flowering habit implying that pollinations can be made all year^2^. The inflorescence develops in the pseudostem after which leaves are replaced by bracts. The first three or four bracts are the largest and typically do not bear flowers. After emergence, the inflorescence takes on a horizontal or pendent position for edible bananas. Female flowers emerge before male flowers and are usually separated by neutral flowers^3^. This implies that there is no self-pollination within an inflorescence except in some genotypes with hermaphrodite flowers^4^. Geitonomous pollination can occur between flowers in different generations on the same mat.

Each bract bears two rows of flowers in a cluster, one above the other. Similar to most monoecious plants, bananas produce more male than female flowers. The flowers are irregular with a compound tepal, androecium and gynaecium as the three main parts. These flower parts are all joined at the point of connection of the style with the ovary thus form an inferior ovary^5^. Depending on genotype, soil fertility, and environmental conditions, the inflorescence usually bears between 1 to 30 female flower clusters, followed by 0 to 4 neutral flower clusters and up to 300 male flower clusters^1^.

Rapid conventional improvement of bananas is hindered by a complex array of factors that result in male and female sterility^6^. Among factors that influence seed set especially for controlled pollination is pollinating at the right time of the day to ensure maximum seed set^7^. Flowers must be pollinated soon after opening as ovules start to disintegrate 24 h after anthesis^1^. On the other hand, pollen viability is highest at 08:00 h and lowest at 16:00 h^8^. Shepherd^7^ obtained the highest seed set in pollinations made at 07:00 h and the lowest after pollinating at 16:00 h. A thorough understanding of banana floral biology could therefore contribute toward solving the poor seed set problem in edible banana. Increase in seed set per cross made would create a wider progeny base for breeders to make meaningful selections and consequently efficient breeding pipelines^6^.

Flower opening time is a tightly regulated trait in plants, and this determines when and which pollinators are involved in the pollination process. This in turn determines the fitness of a plant species to survive. Banana is a facultative day long plant with long photoperiods leading to early inflorescence initiation^9^. Bract opening at night implies that bats participate in pollination, although diurnal insects have also been reported to visit flowers^10^. The exact start of bract opening time (BOT) is unclear in banana, as opposed to crops such as rice, where flower opening time is between 09:00 and 14:00 h^11^. A case study by Amah et al.^6^ found that bract lift in Mchare bananas in Arusha Tanzania started late afternoon but appearance of flowers was after night fall. The study also found that there is maximum flower visitation frequency at dawn which is believed to be the period of maximum stigma receptivity.

Maximum seed set was obtained after controlled pollination of ‘Gros Michel’ between 07:00 and 10:00 h^7^ and this has been widely adopted^6,7^. In rice, flower opening time is known to be controlled genetically by about three genes, but weather conditions have also been observed to have an influence^11^. Weather is likely to trigger flower opening so that pollination happens under favourable night conditions. Since the widely adopted pollination time range is between 07:00 to 10:00 h, the assumption would be no differences in BOT among banana genotypes. Also, female and male flowers are separate in banana and there is no information on whether opening is synchronized on separate plants. The aim of this study was therefore to determine BOT in banana under field conditions and how BOT is influenced by weather conditions.

## Materials and methods

### Field site and banana genotypes used

The experiment was conducted at two sites in Uganda; the National Agricultural Research Laboratories (NARL) in Kawanda and the International Institute of Tropical Agriculture (IITA) Sendusu station, Namulonge. Kawanda is located at 0° 25′ N and 32° 32′ E at an elevation of 1177 m, while Namulonge is located 0° 31′ N and 32° 36′ E at an elevation of 1160 m. Banana genotypes used included *Musa* (AAA-EA group Matooke subgroup) ‘Enzirabahima’, ‘Nakitembe’, ‘Enyeru’, ‘Kabucuragye’^12^, and ‘NARITA 17’ (AAA) which is a Matooke hybrid from collaborative breeding efforts between NARL and IITA^13^. Also used were *Musa* (AA group subgroup Mchare) ‘Mlelembo’ and ‘Kamunyilya’ and *Musa* (AAB group subgroup Plantain) ‘Gonja’ for a B-genome representation. Letters “A” and “B” in groupings denote contributions from progenitors *Musa acuminata* and *M. balbisiana* respectively. Wild banana *M. acuminata* spp. *burmannicoides* ‘Calcutta 4’ was also included.

At NARL, ‘Enzirabahima’, ‘Nakitembe’, ‘Mlelembo’, ‘Kamunyilya’, and ‘Calcutta 4’ were studied between July 25 and September 27, 2016 whereas ‘Enyeru’, ‘Kabucuragye’, ‘NARITA 17’, and ‘Gonja’ were studied at IITA between September 20 and November 11, 2018. Observations were made on already established plants in pollination blocks planted at a spacing of 3 × 2 m. For each genotype, a single inflorescence was studied. Cultivars in the Matooke and Mchare subgroups were treated as replicates since variability within these subgroups are said to have arisen from somatic variation^14,15^. The subgroups studied were therefore; Mchare (2), Matooke (4), Matooke hybrid (1), plantain (1), and wild banana (1).

### Time-lapse photography for bract opening time

Because banana plants usually grow to a height of more than 3.0 m, a table of 1.0 m was used to supplement the camera tripod stand which could go up to a 2.0 m height. The tripod stand was firmly bound on the table surface to avoid movement and shaking of the camera. A Nikon D810 camera (Nikon Corporation, Tokyo, Japan) was used to take photographs of the growing banana inflorescences at an interval of 5 min. The camera flash was set to manual mode with highest power and fastest sync speed; it was left on throughout the study period. The period of flower study started when the inflorescence was in the erect position and pictures were taken continuously until opening of a few bracts subtending male flowers.

With MatLab Version 9.4 (R2018a) software developed by MathWorks (https://www.mathworks.com/), time-lapse videos were made at a speed of 30 frames per second (fps). The videos were played using Kinovea Version 0.8.15.0 software (https://www.kinovea.org/) developed by Joan Charmant and time for bract opening events were recorded. Bract opening events included time of bract lift, time of start and time of end of bract rolling. Bract lift implied the loosening of the bract due for opening on the tightly packed flower bud while start of bract roll time was when the tip of bract started rolling backwards. Bract roll rate was determined by subtracting time of end of bract rolling from time of start of bract rolling. Total time of bract opening events was calculated by subtracting time of end of bract rolling from bract lift time. Events time for each bract were converted to fractions (hours) and averaged separately for bracts subtending male and female flowers for each inflorescence. Data were subjected to one-way analysis of variance without blocking with subgroups as treatments; data for bracts subtending female and male flowers were analysed separately. Total time of bract opening events for bracts subtending female and male flowers was also compared in a paired t-test with genotypes constituting pairs. Analysis of variance and the t-test were run using Genstat *for Windows* 19th edition (http://www.genstat.co.uk) developed by VSN International (VSNi).

Irrespective of when the bract started to open, time-lapse videos were paused at 08:00 h for each bract to measure the angle of bract lift with reference to the rachis for both female and male flowers. Bract roll was scored on the scale of 1 for minimal roll to 5 for complete bract roll. Bracts events that could not be observed for partially or fully obscured bracts were recorded as missing data. Time duration between bracts subtending female and male flower opening was calculated as time of lift of first bract subtending male flowers minus bract roll end-time of last bract subtending the female or transition flower cluster. Average bract roll scores and lift angles per cluster position were plotted against bract position number. For genotypes whose single bract events happened before and after 00:00 h, total hours from 00:00 h of the previous day were counted and averaged.

### Weather influence on bract opening time

Weather data were obtained from the NARL and the Namulonge agro-metrological stations. The Namulonge agro-metrological station is 1.5 km from the IITA – Sendusu station thus data were used for the latter station because of their proximity. From the NARL station, we obtained temperature (°C) data recorded at 15:00 h whereas from the Namulonge station, we obtained average daily temperature (°C) and light intensity (lux). During the study period, temperature at 15:00 h at NARL ranged from 23.5 to 32.0 and averaged 29.0 °C. On the other hand, daily temperature at Namulonge was in the range of 20.5 and 24.6 with an average of 22.8 °C while light intensity ranged between 1167.7 and 3316.2 with an average of 2263.1 lux. Correlation analysis was performed between bract opening events time and weather data. The analysis was performed with Genstat *for Windows* 19th edition (http://www.genstat.co.uk) software developed by VSNi.

### Policy and plant use guidelines

The authors confirm that the banana genotypes used in this present study was in accordance to international, national and/or institutional guidelines.

## Results

### Bract opening events

Banana bract opening events were generally initiated by bract lifting followed by bract rolling (Table 1). In some cases, bract lifting and bract rolling were simultaneous events, especially for the first three female clusters and particularly for ‘Mlelembo’. In observed subgroups, Matooke had the earliest lift and start of rolling of bracts while wild banana ‘Calcutta 4’ lifted and started rolling its bracts last (Table 1). Bract lift and start of rolling was statistically significant for bracts subtending female flowers but not for those of male flowers. And end of bract rolling happened first in Matooke and last in Plantain; this was statistically significant for both bracts subtending female and male flowers.

**Table 1:** Time of day and duration of bract opening events in selected banana genotypes.

Subgroup/genome	Reps	Flower sex	No. of bracts	Days to open	Average time of day (24 h format)			Average time (h:m)	
					Bract lift	Bract roll start	Bract roll end	Bract rolling	Opening
Wild (AA)	1	Female	6	4	20:19	01:09	06:01	4:52	9:17
		Male	10	7	01:03	02:50	06:31	3:41	5:28
Mchare (AA)	2	Female	14	8	16:10	16:57	22:27	7:32	7:59
		Male	3	3	18:21	*	*	*	*
Matooke (AAA)	4	Female	25	16	14:54	15:56	01:23	9:35	10:38
		Male	14	15	16:53	19:01	21:54	3:25	5:02
Matooke Hybrid (AAA)	1	Female	14	8	16:59	18:11	01:10	8:00	9:11
		Male	14	10	19:51	21:56	00:52	2:52	5:44
Plantain (AAB)	1	Female	7	8	19:19	04:45	12:18	7:33	15:59
		Male	2	3	21:00	*	09:52	*	13:40
Average		Female	7.3	4.9	16:32	18:51	02:33	8:12	10:19
		Male	4.8	4.2	18:54	20:48	01:16	*	6:19
F-probability		Female			0.004	<0 .001	0.048	0.164	0.115
		Male			0.078	0.152	0.028	*	0.039
l.s.d (h:m)		Female			1:57	1:31	7:58	4:12	5:54
		Male			5:56	9:59	7:30	*	5:31

*Missing data, l.s.d—least significance difference, Reps—replicates which are number of cultivars within a subgroup, No. of bracts—number of bracts (total bracts observed for a given genotype/subgroup and sex of the flowers). Days to open are total number of days taken for observed bracts to open. Average time of bract events is in hours: minutes. Letters “A” and “B” in the parenthesis after genotype names are genome contributions from progenitors *Musa acuminata* and *M. balbisiana* respectively.

On average, lift and start of rolling events for bracts subtending female flowers happened before events for bracts subtending male flowers, the exception was end of bract rolling. Bract rolling rates among female flowers were not statistically significant, results for male flowers were not analysed as there were many data missing. Total duration of bract opening was not significant among female flowers but was significant among male flowers. Total duration of bract opening events of female flowers was more than that of male flowers (paired T-test prob. > 0.001, 6 d.f.).

**Figure 1:**
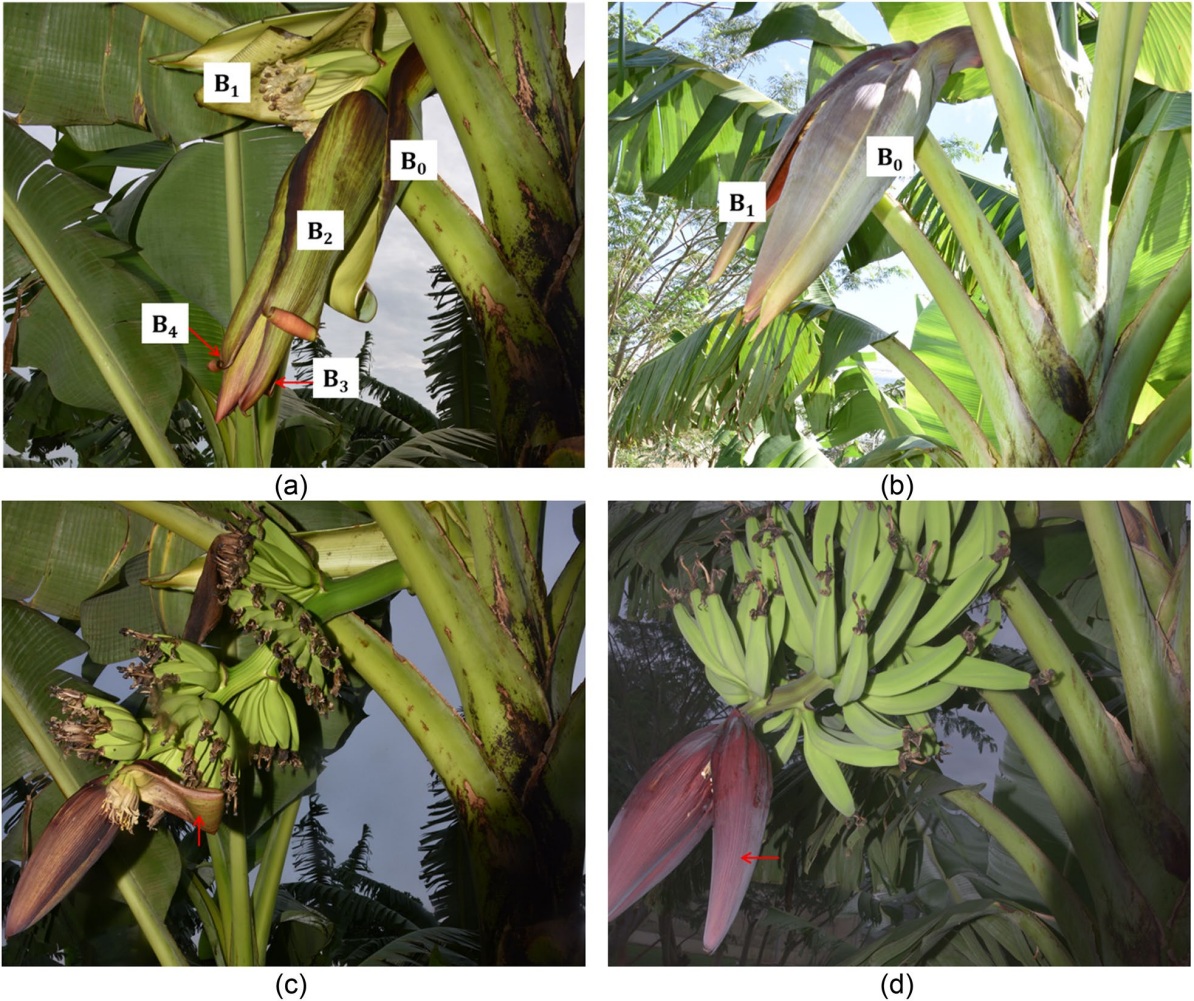
(**a**) ‘Kabucuragye’ (AAA) at 08:03 h after opening of the first bract subtending female flowers. **B**_**0**_ is a non-flower bearing bract. **B**_**1**_ is an almost fully rolled bract subtending the first cluster of female flowers on day one of bract opening. **B**_**2**_ is a partially rolled second bract subtending female flowers before fully opening on day two. **B**_**3**_ obscured but partly visible third bract subtending female flowers with partially rolled bract tip. **B**_**4**_ is the fourth bract subtending female flowers with the tip partially rolled to a less degree compared to former bracts. (**b**) ‘Gonja’ (AAB) at 16:20 h after opening of the first bract subtending female flowers. **B**_**0**_ is one of the non-flower bearing bracts that did not roll. **B**_**1**_ first bract subtending female flowers that did not roll after lifting. Subsequent bracts did not partially roll before opening on the next day. (**c**) Opening of first bract subtending male flowers (red arrow) in ‘Kabucuragye’ at 08:01 h. (**d**) Opening of first bract subtending male flowers (red arrow) in ‘Gonja’ at 08:04 h.

With the exception of ‘Gonja’, all observed inflorescences exhibited a tendency of partial rolling of bracts subtending female flowers a day or two before actual lift and roll (Fig. 1a,b). This happened to a varying degree from proximal to distal end of the inflorescence in different genotypes, especially in the first three to four bracts. On the other hand, bracts subtending male flowers did not exhibit partial rolling of bract tips before fully lifting and rolling in subsequent days (Fig. 1c,d). It was observed that bracts that had not completed rolling and curling of fingers backwards were halted when day broke. The process continued on subsequent days simultaneously with lifting and rolling of fresh bracts. By the time female and male flowers were visible, the compound and free tepals had already opened to expose female and male flower parts in all genotypes studied. In some genotypes especially ‘Enyeru’ and ‘Kabucuragye’, inflorescences moved from the horizontal-pendent position towards the horizontal position at dusk and fell towards the pendent position by mid-morning. This was a repetitive process during bract opening period of female flowers.

**Figure 2:**
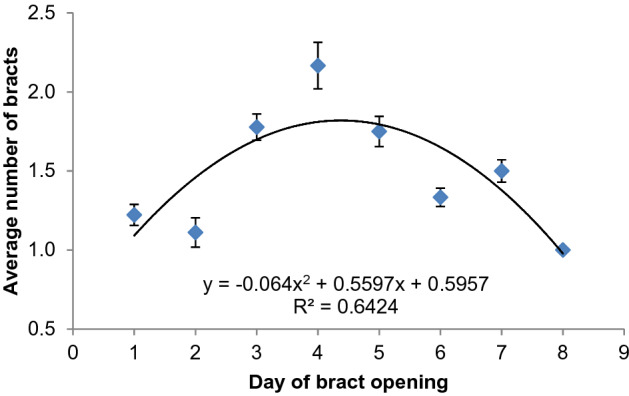
Average number of bracts subtending female flowering opening per day during opening period of selected inflorescences of banana genotypes; error bars denote mean ± 10% standard deviation.

On average, more bracts subtending female flowers opened per day in the mid-section of the inflorescence compared to proximal and distal ends (Fig. 2). One bract subtending female flowers opened on the first day with maximum number of bracts opening on day four. This gradually reduced up to one bract opening on day eight which was the last day of bract opening for female flower clusters. All bracts subtending female flowers opened in a period of three to eight days (Table 1). In the event of multiple bracts opening on the same day, former clusters had flowers with brownish stigmas while latter clusters had flowers with creamy stigmas. And for multiple bracts opening on the same day, opening events were simultaneous or were separated by up to four and a half hours with former bracts opening first. After opening of bracts subtending female and transitional clusters, there was on average a longer lapse before the onset of opening of bracts subtending male flowers. The longest lapse period was in ‘Mlelembo’ while ‘Kamunyilya’, ‘Enzirabahima’, and, ‘NARITA 17’ had lapses of less than a day (Table 2). Lapse duration of less than a day between male and female flowers was the same as the opening interval of bracts subtending female flowers.

**Table 2:** Duration between female and male flowers opening.

Genotype/genome	Days	Hours	Min
‘Calcutta 4’ (AA)	1	14	0
‘Mlelembo’ (AA)	7	*	*
‘Kamunyilya’ (AA)	0	20	7
‘Enzirabahima’ (AAA)	0	14	56
‘Enyeru’ (AAA)	1	16	51
‘Kabucuragye’ (AAA)	3	*	*
Nakitembe (AAA)	3	12	48
‘NARITA 17’ (AAA)	0	18	20
‘Gonja’ (AAB)	5	20	49

*Precise time could not be computed.

### Bract appearance at time of pollination (08:00 h)

**Figure 3:**
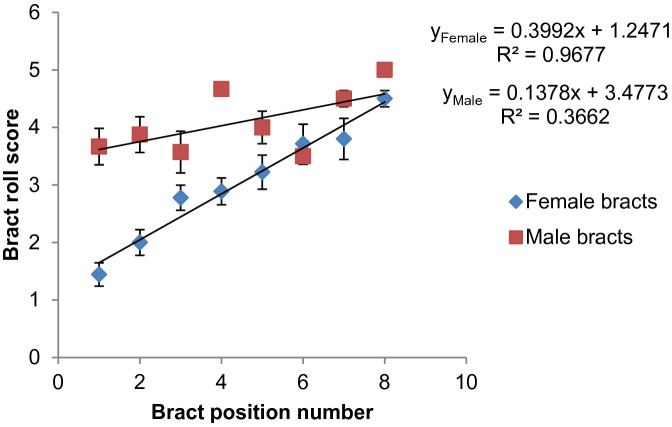
Average bract roll scores of bracts subtending female and male flowers for various inflorescences of selected banana genotypes. A score of 1 = minimal roll and score of 5 = full bract roll, error bars denote mean ± 10% standard deviation.

**Figure 4:**
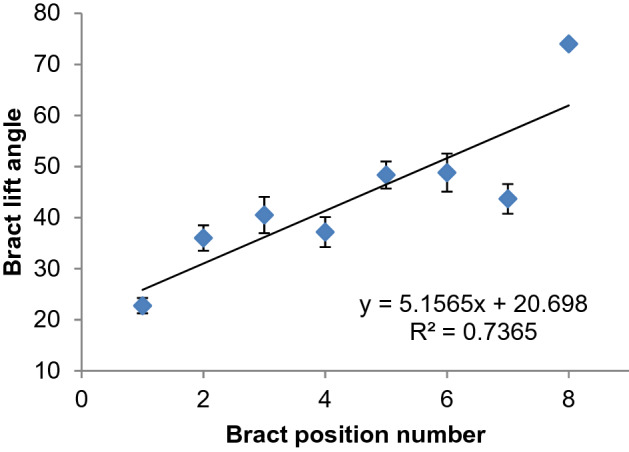
Average bract lift angle of bracts subtending female flowers for different bract positions of 9 selected banana genotypes, error bars denote mean ± 10% standard deviation.

Bracts subtending female flowers at the proximal end of the inflorescence partially rolled, the degree of rolling gradually increased towards the distal end (Figs. 1a, 3). Bracts subtending female flowers at the distal end completely rolled, which was comparable to bracts subtending male flowers. On the other hand, bracts subtending male flowers had a relatively similar appearance after rolling irrespective of the bract position (Fig. 3). The angle of bract lift at 08:00 h was also small in proximal clusters but gradually increased towards the distal end of the inflorescence (Fig. 4). This was generally exhibited by all observed inflorescences. On average, bracts subtending female flowers in position one lifted to about 25° while bracts in position eight lifted to more than 60° by 08:00 h. Male flowers were observed to have more insect visitors compared to female flowers especially between dawn and about 08:00 h.

### Influence of weather on bract opening

Average daily temperature had no significant relationship with time of bract opening events for both female and male flowers at the IITA—Sendusu station (Table 3). There was also no relationship between temperature recorded at 15:00 h and bract opening events time at NARL station (data not presented). High light intensity led to late bract lift, start of rolling and end of rolling among bracts subtending female flowers (Table 3). High light intensity also led to a faster rate of rolling for bracts subtending female flowers, there was no relationship with total events time. On the other hands, high light intensity led to an early lift of bracts subtending male flowers.

**Table 3:** Correlation coefficients between weather attributes and bract opening time in banana at the IITA—Sendusu station.

Bract opening event	Female flowers			Male flowers		
	Temp	Light intensity	N	Temp	Light intensity	N
Lift time	0.302	0.386*	31	− 0.2683	− 0.434*	22
Start of roll	0.337	0.496**	27	0.0441	− 0.121	16
End of roll	0.333	0.374*	29	− 0.2072	− 0.029	21
Roll rate	− 0.249	− 0.395*	27	− 0.1596	− 0.281	16
Events time	0.194	0.186	28	− 0.3776	− 0.189	21

*P < 0.05, **P < 0.01, Daily average light intensity (lux), Temp—Daily average temperature (°C), and N—number of observations.

## Discussion

In banana, bract opening behavior depends on the time of the day, the position of the bract, and sex of the flowers enclosed by the bract. Bract opening is a continuous process especially in the first bracts subtending female flowers of some genotypes; it starts in the evening and continues through the night (Table 1). In cases where bracts did not fully open, the process was halted early morning and resumed in the evening. It is therefore not obvious to judge whether such bracts have opened or not. However, opening is permanent as opposed to some plant species which open and close their flowers at specific times. Ssebuliba et al.^16^ considered East African Highland bananas ready for pollination when bracts were half way open with stigmas having a creamy white appearance. According to observations made in the current study, it can be said that bract lifting is indicative of flower opening thus pollination can start.

Bract lift and bract roll seemed to be a response of a certain light quality^6^, the response time and speed are genotype dependent. Finger curling also seems to be triggered by the same factors that lead to bract opening. Bract opening and finger curling are likely to be a response of changes in turgor pressures in cells that lead to tissues being pushed in a given direction^17^. This was evident with upward movement of the inflorescence from the horizontal-pendent toward the horizontal position in the evening and downward movement towards the pendent position by mid-morning. These movements were genotype dependent and small, maximum oscillation was about 10˚. A similar pattern was observed for leaf folding to influence relative canopy cover^18^.

Generally, bracts subtending female flower lifted and started rolling earlier than those subtending male flowers. However, male flowers ended opening before female flowers, resulting in faster bract opening for male flowers (Table 1 and t-test). This might be due to the smaller bract size of male flowers (Fig. 1) or an adaption for female flowers to find male flowers open with ready pollen. Consequently, the strategy ensures maximum pollination success and survival of the *Musa* spp. Studies have revealed that pollen viability reduces with time after flower opening^1^. This is in agreement that controlled pollination should be done between 07:00 and 10:00 h^7^. In comparison to lilies, some flowers were observed also to open starting at 17:00 h while others open during day. Both nocturnal and diurnal pollinators were found to be active flower visitors^19^. This implies that pollination in banana can start in the evening as long as bracts for parents in the cross of interest lift in time.

In *Musa itinerans*, two nectar production peaks were found, that is between 08:00 to 12:00 h and 20:00 to 24:00 h^20^. This maybe a close depiction of what happens in edible bananas thus emphasizing the potential importance of diurnal and nocturnal pollinators. Bats, bees, and birds were found to be among the most important pollinators of bananas at Onne, Nigeria^10^. However, natural pollinators were not the main focus of the study though they are good indicators of when stigmas might be highly receptive. Since nectar quality and quantity varies between different agro-ecologies and seasons^21^, flower visitations and seed set are also expected to vary accordingly. Different agro-ecologies are also expected to experience variable BOTs due to variable solar radiation. Likewise the different growing seasons (rainy and dry) might also affect BOTs and therefore seed set^22^. However, a comparison of time from sunrise to beginning of bract lift of *Musa* AAA Cavendish cultivars in a glasshouse and *M. basjoo* in the garden in Belgium revealed no significant difference^6^. But comparison of bract curling time in Mchare in Arusha with short days and Cavendish cultivars in a glasshouse in Belgium with long days in summer, there was early curling in the glasshouse. However, bract lift time may be a better event to use for comparison than bract curling or rolling time.

Bracts of both female and male flowers of different genotypes completed opening at different times and this may be partly the reason for variable pollen viability and stigma receptivity (Table 1). Female flowers that finish opening much earlier may set less seed compared to those that finish opening closer to the routine time of hand pollination between 07:00 and 10:00 h. Conversely, male flowers that are ready shortly before the time of hand pollination are expected to have higher pollen viability. This probably explains the high fertility of ‘Calcutta 4’ as it finished opening at 06:30 h. Some male flowers like those of Matooke finished opening as early as 21:54 h (Table 1) and are expected to have pollen with low viability at the time it is measured the next day.

All observed inflorescences opened one female bract on the first day, increasing to multiple bracts opening on subsequent days (Fig. 2). One to three bracts subtending female flowers were observed to open per day from the second bract position of the inflorescence. The pattern of opening took on a hyperbolic shape with up to four bracts opening on the fourth day in the midsection of the inflorescence. For hand pollination, more clusters are therefore expected to be pollinated per day during bract opening in the mid-section of the inflorescence. The different clusters of female flowers that open on the same day are likely to have stigmas with varying receptivity. The darker appearance of stigmas of former clusters compared to creamy stigmas in latter clusters reflects higher receptivity in the latter^2^. This may explain why some clusters set more seed especially in the mid-section of a seemingly fertile inflorescence.

Upon complete opening of female and transitional bracts, inflorescences went into a pause period before male flowers opened (Table 2). In additional to spatial separation of flowers, this is temporal separation to promote cross pollination in banana. However, temporal separation of male and female flowers is not very effective for genotypes that had less than 24 h of separation. With aid of crawling insects, self-pollination may happen between the last female cluster and the first male cluster as stigmas are likely to be receptive for more than one day. Once male flowers started opening, one bract opened per day and occasionally two bracts were observed to open on the same day. For highly fertile genotypes like ‘Calcutta 4’, ample pollen is produced to pollinate many female flowers. Male flowers are also produced throughout the inflorescence growth period which ensures constant supply of pollen especially for controlled hand pollination. Averages of bracts subtending male flowers opening per day could not be calculated as there were two to three observed bracts subtending male flowers for most genotypes.

It appears that proximal bracts subtending female flowers are less stimulated to lift and roll compared to distal bracts subtending female flowers and all bracts subtending male flowers. This was revealed by low vigour of bract lift and the small angle of lift at 08:00 h especially in the first female flower cluster (Figs. 2, 3). The bract angle of lift increases from proximal to distal end and this has been linked to reduced fertility in proximal clusters^2^. But it may not be the case since highly female (in all clusters) and male fertile ‘Calcutta 4’ showed the same pattern as edible bananas. The high R^2^ for female bract roll scores compared to bracts subtending male flowers was a result of more bracts used to calculate averages for bracts subtending female flowers compared to bracts subtending male flowers (Fig. 3). For bracts subtending male flowers, two to three bracts were observed for most genotypes thus the first three data points were close to the trend line. Since the number of female clusters varies, reducing number of data points were used to calculate average bract lift angles in the distal end or larger inflorescences. Besides, bract lift angles of some clusters could not be measured because of obscurity or being in awkward positions. This led to the last two points being far off the trend line for angle of lift and hence a low R^2^.

Flower opening time is said to be genetically and environmentally controlled, results from this study are in agreement since light had considerable influence on bract opening events (Tables 1, 3). Significant effects of temperature, solar radiation, and vapor pressure deficit on flower opening time have been observed in rice^11^. For *Musa* spp., only light has a significant relationship with BOT. However, there was early curling under long summer days in the glasshouse in Belgium compared to short days in Arusha field conditions^6^. This suggested a particular light signal for BOT in *Musa* spp. It is unclear why high light intensity led to early lift of bracts subtending male flowers and this calls for farther investigation. Since bracts subtending male flowers instinctively open later than bracts subtending female flowers, light intensity had less effect on the former bracts. The small sample size could have also had an impact on the results in the study, the light flush from the camera could have also affected the results. The extent of weather effects on BOT in banana need to be studied in field conditions of locations with significantly different day length for a more reliable conclusion.

## Conclusion

Hand pollinations in banana have to be done at the right time for maximum seed set which is critical for their improvement. This study assessed BOT to determine when flowers are likely to be most receptive. Banana bracts subtending female flowers start lifting late afternoon and complete opening mostly after midnight. This implies that current controlled pollination at 07:00 to 10:00 h might perhaps be too late. But it would make sense to try and pollinate as early as possible especially as soon as bracts subtending male flowers open. The aim should also be to pollinate when pollen viability is highest just after bract opening considering the fact that opening events time for bracts subtending female and male flowers may be different. Since bananas open bracts partly during day and partly at night, nocturnal and diurnal pollinators have a role to play as natural pollinators. Bract rolling and lift angles seem not to be linked to fertility as the highly fertile wild banana ‘Calcutta 4’ behaved just like the sterile edible bananas. For some genotypes like ‘Gonja’, there is a considerable time lapse between female and male flower opening thus self-pollination is not a concern with aid of pollinators.

## Data Availability

Data available upon reasonable request.

## Abbreviations

BOT: Bract opening time
NARL: National Agricultural Research Laboratories
IITA: International Institute of Tropical Agriculture
AA: Diploid group of bananas with genome contribution from *Musa acuminata*
AAA: Triploid group of bananas with genome contribution from *Musa acuminata*
AAB: Triploid group of bananas with genome contribution from *Musa acuminata and Musa balbisiana* designated as A and B respectively
fps: frames per second
R^2^: R-squared which is a goodness-of-fit measure for linear regression models
